# Genomic and Phenotypic Insights Into the Potential of Rock Phosphate Solubilizing Bacteria to Promote Millet Growth *in vivo*

**DOI:** 10.3389/fmicb.2020.574550

**Published:** 2021-01-07

**Authors:** Ubiana C. Silva, Sara Cuadros-Orellana, Daliane R. C. Silva, Luiz F. Freitas-Júnior, Ana C. Fernandes, Laura R. Leite, Christiane A. Oliveira, Vera L. Dos Santos

**Affiliations:** ^1^Department of Microbiology, Universidade Federal de Minas Gerais, Belo Horizonte, Brazil; ^2^Centro de Biotecnología de los Recursos Naturales, Universidad Católica del Maule, Talca, Chile; ^3^Grupo de Genômica e Informática de Biossistemas, Centro de Pesquisas René Rachou, Fiocruz, Belo Horizonte, Brazil; ^4^Embrapa Milho e Sorgo, Sete Lagoas, Brazil

**Keywords:** bacteria, phosphate, solubilization, maize, plant growth promotion

## Abstract

Rock phosphate (RP) is a natural source of phosphorus for agriculture, with the advantage of lower cost and less impact on the environment when compared to synthetic fertilizers. However, the release of phosphorus (P) from RP occurs slowly, which may limit its short-term availability to crops. Hence, the use of P-solubilizing microorganisms to improve the availability of P from this P source is an interesting approach, as microorganisms often perform other functions that assist plant growth, besides solubilizing P. Here, we describe the characterization of 101 bacterial isolates obtained from the rhizosphere and endosphere of maize plants for their P solubilizing activity *in vitro*, their growth-promoting activity on millet plants cultivated in soil amended with RP, and their gene content especially associated with phosphate solubilization. For the *in vitro* solubilization assays, two mineral P sources were used: rock phosphate from Araxá (Brazil) mine (AP) and iron phosphate (Fe-P). The amounts of P released from Fe–P in the solubilization assays were lower than those released from AP, and the endophytic bacteria outperformed the rhizospheric ones in the solubilization of both P sources. Six selected strains were evaluated for their ability to promote the growth of millet in soil fertilized with a commercial rock phosphate (cRP). Two of them, namely *Bacillus megaterium* UFMG50 and *Ochrobactrum pseudogrignonense* CNPMS2088, performed better than the others in the cRP assays, improving at least six physiological traits of millet or P content in the soil. Genomic analysis of these bacteria revealed the presence of genes related to P uptake and metabolism, and to organic acid synthesis. Using this approach, we identified six potential candidates as bioinoculants, which are promising for use under field conditions, as they have both the genetic potential and the experimentally demonstrated *in vivo* ability to improve rock phosphate solubilization and promote plant growth.

## Introduction

Phosphorus (P) is an important macronutrient for plant development due to its structural role in the synthesis of nucleic acids and membranes. Furthermore, it plays an essential role in practically all processes involving energy transfer, such as photosynthesis; hence, an adequate P supply is required for plant growth, and its bioavailability in soil can influence crop productivity (Bisson et al., 2017). In maize, P is required from early development (i.e., from germination to 35–40 days), through the flowering and grain-filling stages, which are the most dependent on this nutrient (Coelho and Alves, 2003). However, when P is added to soil in the form of soluble fertilizers, it rapidly forms insoluble complexes with calcium in alkaline soils, and with iron, aluminum and silicates in tropical acidic soils (Novais and Smyth, 1999). This process makes soil P less available to plants, thereby affecting their productivity (Devau et al., 2009).

The search for technologies to improve phosphorus use efficiency has been continuously encouraged. In this context, the use of rock phosphate (RP) is a promising alternative, due to its residual effect in the soil, which increases the P content over time. RPs also have lower cost and generate less environmental impact when compared to more soluble fertilizers, as they contribute much less to the eutrophication of surface waters (Carpenter et al., 1998). Yet, these phosphates have lower reactivity when compared to the highly soluble synthetic fertilizers. In order to compensate for this drawback, several authors have evaluated the simultaneous application of RP and solubilizing microorganisms to the soil, as a way to increase the availability of P from this less soluble source (Ribeiro et al., 2018; Yagi et al., 2020).

Phosphorus-solubilizing microorganisms include both bacteria and fungi (Silva et al., 2014; Mendes et al., 2015; Abreu et al., 2017; Satyaprakash et al., 2017). The main mechanism associated with P solubilization is the release of organic acids during growth (Mendes et al., 2013). The hydroxyl and carboxyl groups of organic acids acidify the surrounding medium, form complexes and chelate cations present on the RP structure, releasing soluble P (Sagoe et al., 1998). In media with high Al and Fe content, organic acids form organometallic complexes, which decrease the toxicity of these elements (Jarosz-Wilkolazka and Gadd, 2003). Moreover, the release of H^+^ ions during NH_4_^+^ assimilation or through other metabolic reactions that trigger proton excretion, such as respiration, could also result in P solubilization (Illmer and Schinner, 1995). Thus, the synthesis of organic acids gives competitive advantages to the producer microorganism on P acquisition at the same time as it benefits other biotic components of the system, including plants.

P solubilization is among the main mechanisms through which microorganisms can promote plant growth, even though microbes are also acknowledged for assisting plant growth and development in multiple ways. Some of them are nutrient (e.g., nitrogen and iron) acquisition, the production of phytohormones and volatile organic compounds (VOCs) and, more indirectly, the control of phytopathogens and the induction of plant resistance (Silva et al., 2016). Research on plant growth-promoting bacteria (PGPB) has been encouraged in view of their use as bioinoculants for agriculture. In addition to stimulating plant growth, it is desirable for the bioinoculants to be good competitors and to be well adapted to the rhizospheric and/or endophytic environment. Some P-solubilizing bacteria have been launched as commercial inoculants, such as QuickRoots^®^ by AcceleronSAS (Saint Louis, United States), and BiomaPhos^®^, the first Brazilian biological product in this line, which was released by Embrapa Maize and Sorghum (EMS, Brazil) in partnership with Bioma (Paraná, Brazil). The BiomaPhos^®^ bioinoculant contains a consortium of two *Bacillus* strains selected by EMS, namely *B. megaterium* CNPMS119 and *B. subtilis* CNPMS2084, both efficient in phosphate solubilization (Abreu et al., 2017; Ribeiro et al., 2018; de Sousa et al., 2020).

The selection of candidate bioinoculant bacteria with diverse plant growth promoting phenotypes has been carried out using *in vitro* tests under laboratory conditions. Although several studies have verified the effectiveness of the inoculation of microorganisms obtained by this approach on the growth and productivity of plants, including maize (de Sousa et al., 2020; Pereira et al., 2020), the poor reproducibility of laboratory assays in plant-soil systems in field scale is still a difficulty that needs to be overcome. It has been suggested that the efficiency of inoculation varies according to the type of soil, cultivar and environmental factors, and microbial characteristics (Silva et al., 2016). In this context, metagenomic and genomic analyses can shed light on the modulation of microbial communities under different cultivation conditions and help to predict the nature of the phenotypic features regarded as important for plant growth, as well as the possible pathways involved.

In a previous study, metagenomics allowed us to check the enriched groups in an area with a long-term history of maize cultivation in soil fertilized with RP in comparison to those cultivated in soils fertilized with Triple Superphosphate (TSP) or in unfertilized soils of the EMS farm (Silva et al., 2017). The differentially abundant taxa in RP-fertilized sites were *Klebsiella* (Enterobacteriaceae), *Massilia* and *Herbaspirillum* (Oxalobacteraceae), *Burkholderia* sp. (Burkholderiaceae) and *Bacillus* sp. (Bacillaceae). We hypothesize that these microorganisms contribute to an increase in P availability in RP-fertilized soil, which promoted plant growth, as maize plants grown in this site showed similar growth to those cultivated in TSP-added soil. Therefore, we believe that this site could be a good source of RP-solubilizing bacteria. Many of the enriched bacterial groups were isolated through traditional approaches in studies conducted in the area (Abreu et al., 2017; Ribeiro et al., 2018), and were deposited in cultures of EMS and UFMG. Among these are the bacteria included in the commercial product BiomaPhos^®^.

Thus, in this study we aimed to: (1) obtain bacterial isolates from the rhizosphere of maize plants in soil fertilized with RP and soil without P fertilization; (2) determine the *in vitro* potential of these isolates, together with that of other rhizospheric and endophytic bacteria previously isolated from maize plants of the same EMS farm, for the solubilization of P; (3) evaluate their ability to stimulate millet growth in non-sterile soil fertilized with RP in a greenhouse environment; and (4) investigate the gene pool related to phosphorus solubilization and uptake of six selected strains, in order to better understand the relation between genotype, phenotype and fitness.

## Materials and Methods

### Bacteria Origin and Cultivation Conditions

In this study, 101 bacteria were used, 32 of which were isolated from maize rhizosphere by enrichment technique using the Araxá Phosphate (AP) as P source, described in this study, 2 were also isolated from maize rhizosphere and obtained from the Multifunctional Microorganisms Collection from Embrapa Maize and Sorghum, Brazil (CMMF-EMS), 57 endophytes were previously isolated from maize and belong to the UFMG Applied Microbiology Laboratory (LMA-UFMG) culture collection, and 10 endophytes were also isolated from maize and kindly provided by CMMF-EMS (Supplementary Table S1).

All endophytic isolates evaluated in this study were selected for their potential to solubilize calcium phosphate, which was previously demonstrated in studies conducted by our research group (Vieira et al., 2015; Abreu et al., 2017). The isolates belong to the genus *Bacillus* (15%), *Pantoea* (13.5%), *Serratia* (12%), *Enterobacter* (12%), *Klebsiella* (9%) and *Acinetobacter* (6%). The other genera (i.e., *Arthrobacter, Brevibacillus, Curtobacterium, Erwinia, Flavobacterium, Lactococcus, Microbacterium, Obesumbacterium, Ochrobactrum, Pseudomonas, Raoultella, Rhizobium, Serratia*, and *Staphylococcus)* represent each less than 5% of the total.

### Isolation of Potentially P-Solubilizing Bacteria

For the isolation of potentially P-solubilizing bacteria using the enrichment technique, samples from rhizospheric soil of maize plants cultivated in soil without the addition of P (1) and soil fertilized with AP (2) were inoculated into 125 mL Erlenmeyer flasks containing 50 mL of NBRIP (National Botanical Research Institute) medium (Nautiyal, 1999) plus AP (5 g/L). AP is an apatitic phosphate of igneous origin from a deposit located in the state of Minas Gerais in Brazil. It exhibits a high crystallization level with a small degree of isomorphic substitution of PO_4_^–3^ by CO_3_^–2^ in the apatite crystal that results in low 2% citric acid solubility (Lehr and McLellan, 1972). The physical and chemical characteristics of these P sources are shown in Table 1.

**TABLE 1:** Physical and chemical characteristics of the P sources used in this work.

P source	Total P_2_O_5_ (%)	P_2_O_5_ in CA^1^ (%)	Gran. ^2^ (mm)	Structure	Origin
Araxá Phosphate	30	5.7	0.06	Fluorapatite/some hydroxyapatite	Igneous
Commercial Rock Phosphate	28	9	0.30	Fluorine-hydroxy-carbonate apatite	Sedimentary
Super Triple Phosphate	46	40	_	_	_

*^1^Soluble in 2% citric acid. ^2^Granulometry.*

The flasks were incubated at 28°C, with rotary shaking at 180 rpm for 48 h. After this period, 10 mL of the cultures were transferred to fresh NBRIP medium containing AP (5 g/L) and incubated once more for 48 h under the same conditions. This procedure was repeated a third time and the culture was centrifuged and resuspended in sterile saline solution (0.85% NaCl). Serial dilutions were made, and 100 μL aliquots of the 10^–1^ to 10^–7^ dilutions were inoculated by spreading onto NBRIP + tricalcium phosphate solid medium (selective medium/indicator of P solubilizing bacteria). After 48 h incubation at 37°C, colonies presenting a solubilization halo were considered positive and were stored at −80°C in Tryptone Soy Broth (TSB, Himedia, Mumbai, India) with 15% (v/v) glycerol.

### Molecular Identification of Bacteria Isolated From Maize Rhizosphere Samples by the Enrichment Technique

#### Extraction of Bacterial DNA

The genomic DNA of the bacteria isolated from the enrichment culture was extracted using a protocol defined by Pitcher et al. (1989), with modifications. Bacterial cells were reactivated by striking Soybean Agar Tryptone medium (TSA, Himedia, Mumbai, India) and plates incubated for 24 h at 27°C. Then, individual colonies were grown in TSB liquid medium for 24 h at 37°C. After this period, they were transferred to 2 ml microtubes, centrifuged and washed with 1 mL of saline solution (0.85% NaCl). The pellet was resuspended in 250 μL of TE buffer (Tris-EDTA – Tris-HCl 10 mmol/L, EDTA 1 mmol/L, pH 7.5), added with glass beads and vortexed for 2 min. Four μL of the extraction buffer containing 5.0 mol/L guanidine thiocyanate solution, 100 mmol/L EDTA (pH 8.0) and 0.5% sarcosyl were added. The microtubes were homogenized by inversion and incubated at room temperature for 10 min. After adding 200 μl of cold 7.5 mol/L ammonium acetate, the tubes were shaken by inversion and incubated on ice for 10 min. After this period, 600 μL of chloroform-isoamyl alcohol (24: 1, v/v) were added and the tubes were shaken again by inversion. After centrifugation at 12,000 *g* for 10 min, the aqueous phase was transferred to new microtubes, in which the same volume of chilled isopropanol was added. Then, the microtubes were kept at room temperature for 1 h and centrifuged at 4,000 *g* for 20 min. The supernatant was discarded and the pellet washed with 70% (v/v) ethanol. Then, the samples were centrifuged for DNA precipitation. The tubes were kept open at room temperature until all the ethanol evaporated. At the end, the DNA was diluted in 50 μL of sterile deionized water and stored at −20°C. The product was quantified in NanoDrop ND 1000 (NanoDrop Technologies).

#### Amplification and Sequencing the 16S rRNA Gene Region

Genomic DNA was extracted from the isolates and the V1–V5 or V1–V4 regions of the 16S rRNA gene were amplified using the standard universal primer pairs 8F (5′-AGAGTTTGATCCTGGCTCAG-3′) and 907R (5′-CCGTCAATTCCTTTRAGTTT-3′) or 27F (5′-AGAGTTTGATCMTGGCTCAG-3′) and 806R (5′-GGACTACHVGGGTWTCTAAT-3′), respectively. The PCR reaction contained the following: buffer 1X (20 mmol/L Tris-HCl pH 8.4, 50 mmol/L KCl); 1.5 mmol/L MgCl_2_; 200 μmol/L of each of the deoxyribonucleotides (dATP, dCTP, dGTP, dTTP); 0.5 μmol/L of each primer; 1.5 U of Taq DNA polymerase (Synapse Biotechnology); 50 ng of template DNA in a final volume of 25 μl.

PCR was carried out using a Veriti thermal cycler (Applied Biosystems) under the following conditions: initial denaturation at 94°C for 4 min, 30 cycles of denaturation at 94°C for 1 min, annealing at 57°C for 1 min for the 8F-907R primers or at 52°C for 1 min for the 27F-806R primers, followed by extension at 72°C for 1 min and 30 s; with a final extension of 72°C for 10 min. The amplified products were analyzed by electrophoresis on a 1% agarose gel stained with ethidium bromide to assess their integrity and size, and then quantified in a NanoDrop ND 1000 spectrophotometer (NanoDrop Technologies).

Next, the PCR products were purified using 11.25 μL of EDTA (125 mmol/L) and 135 μL of absolute ethanol (Merck), incubated for 15 min at room temperature and centrifuged at 4,000 *g* for 60 min. The supernatant was discarded, and the pellet was washed with 120 μL of 70% (v/v) ethanol. After ethanol evaporation, the purified DNA was resuspended in 20 μL of sterile deionized water, and quantified in NanoDrop ND 1000. For sequencing, the BigDye^®^ Terminator v3.1 kit (Applied Biosystems^®^) was used, following the manufacturer’s recommendations for the ABI 3700 automatic sequencer.

The sequences were trimmed for quality using the software Bioedit Version 7.2.5 (Hall, 1999) and Asparargin^1^. After removing low quality regions (Phred score < 20), the sequences were compared with the databases, GenBank, SILVA, RDP (Ribosomal Database Project) and Greengenes.

### Evaluation of Potential of Araxá and Iron Phosphates Solubilization by Bacterial Isolates by *in vitro* Tests Under Agitation

Bacterial isolates were grown in 125 mL Erlenmeyer flasks containing 50 mL of liquid NBRIP medium supplemented with AP (5 g/L) or Fe–P (1 g/L). The flasks were inoculated with 1 ml of bacterial suspension adjusted to OD_600_ equal to 1 and incubated at 28°C, 180 rpm for 3 days. After the incubation period, aliquots were removed to assess the concentration of solubilized P by the molybdenum blue colorimetric method at 880 nm (Murphy and Riley, 1962) and the final pH of the culture medium. The final concentration of P was estimated based on the comparison with the standard curve made with KH_2_PO_4_ at concentrations ranging from 0 to 6 mg/L.

### Evaluation of Araxá Phosphate Solubilization Potential by Bacteria in Sessile State

In this test, the bacteria showing the greatest activities in the AP and Fe–P solubilization tests (35 endophytic and 21 rhizospheric bacteria) were used. To prepare the inoculum, the bacterial isolates were activated on the plates containing TSA medium (Soy Triptone Agar) at 28°C for 24 h and resuspended in the NBRIP medium without adding phosphate to a cellular concentration of 0.44 optical density units measured at 600 nm. The tests were performed in 96-well polystyrene plates containing 100 μL of the bacterial inoculum and 100 μL of the NBRIP medium with AP (10 g/L) that was pipetted under constant agitation to avoid phosphate precipitation. The plates were incubated statically at 28°C for 3 days. After the incubation period, the supernatant was transferred to a new plate for the determination of the soluble phosphorus, this culture supernatant was centrifuged at 1,000 *g* for 5 min. Hundred and eighty μL of the supernatant was transferred to a new plate and 40 μL of the reagent mixture was added to measure the solubilized phosphorus, using the molybdenum blue method (Murphy and Riley, 1962). For the determination of soluble phosphorus, after 20 min of incubation the soluble P was quantified colorimetrically at 880 nm.

### Evaluation of Millet Growth in Soil Fertilized With Commercial Rock Phosphate or Triple Superphosphate and Inoculated With Pre-selected Bacteria

For the greenhouse tests, six bacteria were selected from *in vitro* tests. The choice was made in order to have isolates of different genera and from different groups according to the K-means clustering based on P-solubilization. *Microbacterium* sp. UFMG61, *K. variicola* UFMG51 and *Pseudomonas* sp. UFMG81 were chosen due to their greater capacity of solubilize P in the treatments with AP; *P. ananatis* UFMG54 was selected for having a high P-solubilization capacity of both AP and FeP; *B. megaterium* UFMG50 was chosen due to the relevance of this genus for the promotion of plant growth (Zhou et al., 2016; Nascimento et al., 2020); and *O. pseudogrignonense* CNPMS2088 was selected in order to increase the diversity of evaluated genera and because this strain has the ability to increase maize growth parameters in field conditions (data not yet published).

These bacteria were grown in TSB broth for 24 h, centrifuged at 4,000 *g* for 20 min, and the pellet resuspended in sterile 0.85% NaCl solution. The suspensions were adjusted to an optical density of 1.0 at 550 nm, corresponding to 10^8^ cells/mL.

Millet (*Pennisetum glaucum*), variety BRS 1501, was used as a model plant and was cultivated in pots containing 4 kg of typical dystrophic Red Latosol, pH 5.2, 0.4 cmolc/dm^3^ Al, 2.5 cmolc/dm^3^ Ca, 0.2 cmolc/dm^3^ Mg and 30 mg/dm^3^ K, 2.2 mg/dm^3^ P, cation exchange capacity of 11.8 cmolc/dm^3^, base saturation of 23.2% and clay content of 74 dag/Kg.

Approximately 15 days before planting, the soil was limed to correct acidity and a nutrient solution containing macronutrients and micronutrients was added: For 1 L, 22.8 g NH_4_NO_3_, 9.9 g (NH_4_)_2_SO_4_, 30.58 g KCl, 0.23 g H_3_BO_3_, 0.63 g CuSO_4_. 5 H_2_O, 0.74 g MnSO_4_. H_2_O, 0.67 g ZnCl_2_, 0.4 g (NH_4_)_6_Mo_7_O_24_.4H_2_O. The experiment consisted of a factorial of two P treatments and six bacteria strains, as well as an uninoculated control treatment and a control without phosphorus fertilization and inoculation, arranged in a completely randomized design with four replicates.

Two different phosphorus sources were evaluated independently: (1) Triple superphosphate (TSP) and (2) commercial rock phosphate (cRP) of sedimentary origin, which is more soluble source than AP and commercialized in Brazil as reactive natural phosphate (>28% total P_2_0_5_ and 9% P_2_0_5_ soluble 2% citric acid) (BRASIL, 1997) (Table 1).

Both P sources were used at a final concentration of 300 mg P/dm^3^ soil. Prior to all experiments, the millet seeds were prepared with a vehicle aimed at promoting microorganism-seed adhesion, which contained cassava starch and charcoal, as follows: 500 g of seeds were mixed with 500 mL of a cassava starch solution at a concentration of 10% (w/v) and 12.5 g of charcoal. About 20 seeds were sown in pots containing 4 kg of soil at a depth of 3 cm, and then 10 mL of each bacterial suspension were added to each pot. Excess seedlings were removed soon after germination, and 8 plants were maintained in each pot.

During cultivation, the pots were watered daily with distilled water to maintain approximately 80% water field capacity of the soils and 45 days from planting, at the beginning of flowering, the plants were collected. To estimate plant growth based on P sources, we evaluated the dry biomass of both shoot and roots, the average plant height per pot, the average leaf area of plants per pot, the photosynthetic activity on collection day, the content of P in the soil and the accumulation of P in the root and shoot of plants.

### Genomic Analysis of Selected Bacteria

The genomic DNA of the six bacteria selected based on their performance on *in vitro* tests was extracted using the Promega Genomic DNA Purification kit (Madison, United States), following the manufacturer’s recommendations. Then it was sequenced on an Illumina MiSeq platform using the paired-end method with the Nextera XT DNA Library Preparation kit (Illumina, San Diego, CA, United States). The obtained sequences were analyzed for quality and assembled using the A5 protocol (Coil et al., 2014). The resulting contigs were analyzed for completeness using BUSCO v.4.1.2 (Seppey et al., 2019), run on genome mode, setting lineage to the Order level (except for *Microbacterium* sp., to which the lineage was set to Class level) and default parameters. Annotation was done with Prokaryotic Genome Annotation System (PROKKA) (Seemann, 2014) and a modular and extensible implementation of the RAST algorithm (RAST-TK) on the PATRIC platform (Aziz et al., 2008; Brettin et al., 2015). Then, through a textual search, the relationship of coding sequences (CDS) belonging to the P metabolism pathways, production of organic acids and phytohormones was investigated in each genome.

For taxonomic analysis, the genome sequence data were uploaded to the Type (Strain) Genome Server (TYGS^2^), for a whole genome-based taxonomic analysis (Meier-Kolthoff and Göker, 2019). The protein profile of bacterial cultures was analyzed via matrix-assisted laser desorption ionization- time of flight mass spectrometry (MALDI-TOF/MS). A small portion of bacterial biomass cultured in TSA medium was smeared directly onto a stainless steel MALDI sample plate (Bruker, Germany) and covered with 1 μL of formic acid (P.A). After drying at room temperature, the smear was covered with 1 μL of the HCCA matrix (a-cyano-4-hydroxycinnamic acid, Sigma-Aldrich, Poland). After the matrix was dried at room temperature, the plate was inserted into the spectrometer for automated measurement and data interpretation. The mass spectra were processed with the Bruker Daltonik MALDI Biotype software package (Bruker, Germany). The results were obtained from the 20 best identification matches, along with confidence values ranging from 0.00 to 3.00. Values below 1.70 do not allow reliable identification; values between 1.70 and 1.99 allow identification at the gender level; between 2.00 and 2.29 means highly likely identification at the genus level and probable identification at the species level; and a value greater than 2.30 (2.30–3.00) indicates highly likely identification at the species level.

### Statistical Analysis

Phosphate solubilization *in vitro* experiments and the greenhouse tests were arranged in a completely randomized design, with four replications. The averages of the results of the AP and FeP solubilization tests by planktonic cells and AP solubilization tests by sessile cells were grouped by the K-means method using Past (Hammer et al., 2001). The results of P solubilization tests, as well as the parameters for evaluating millet growth were subjected to analysis of variance and the Scott-Knott means comparison test at 5% probability using Sisvar (Ferreira, 2011). Linear correlation analyzes were also performed for the variables P solubilized and final pH of the medium using the R program (R Development Core Team, 2008).

## Results

### Taxonomic Profile of Rhizospheric Bacteria Isolated From Soils With Different Phosphate Contents

Thirty-two bacterial isolates able to solubilize P, as evidenced by the presence of large clear zones around colonies formed in the indicator medium, were selected from two enrichment cultures. The isolates were identified by sequencing a region of approximately 600 bp of the 16S rRNA gene. The isolates obtained from the enrichment culture using rhizosphere soil of maize plants cultivated in soil fertilized with AP (AP-enrichment) belong to the Firmicutes (three isolates) and to the Proteobacteria (Gamma subdivision, 13 isolates), while those from an enrichment using maize rhizosphere soil without any P (control enrichment) are representatives of the Proteobacteria (Beta subdivision, one isolate; Gamma subdivision, 14 isolates) and Actinobacteria (one isolate) (Table 2). *Klebsiella* (10 isolates) was the most frequently recovered genus from the AP-enrichment, corresponding to 62.5%, followed by *Pantoea* (four isolates), *Enterobacter* (one isolate) and *Lysinibacillus* (one isolate). In the control enrichment, *Enterobacter* (seven isolates) was the most frequently recovered genus, followed by Klebsiella (five isolates), Erwinia (one isolate), Pantoea (one isolate), *Burkholderia* (one isolate) and *Curtobacterium* (one isolate). These 32 isolates selected for their P solubilization ability, together with other 69 endophyte and rhizosphere obtained from the LMA (UFMG) and the CMMF-EMS culture collections (Table 2), were further characterized using quantitative assays.

**TABLE 2:** Taxonomic classification of bacterial isolates in the study.

Origin	Phyla/Classes	Genera	Number of isolates
Enrichment culture with rhizosphere soil of maize plants cultivated with AP (This study)	Firmicutes	*Lysinibacillus Bacillus*	1 2
	Proteobacteria/Gammaproteobacteria	*Klebsiella Pantoea Enterobacter*	9 3 1
Enrichment culture with rhizosphere soil of maize plants cultivated without P fertilization (This study)	Proteobacteria/Gammaproteobacteria	*Enterobacter Klebsiella Erwinia Pantoea*	7 5 1 1
	Proteobacteria/Betaproteobacteria	*Burkholderia*	1
	Actinobacteria	*Curtobacterium*	1
Total			32
Endophyte: Collection LMA/UFMG*	Proteobactria (Gamaproteobacteria)	*Serratia Pantoea Enterobacter Klebsiella Acinetobacter Pseudomonas Curtobacterium Obesumbacterium Raoultella Rhizobium*	7 7 7 6 4 2 2 1 1 1
	Bacteroidetes	*Flavobacterium*	2
	Firmicutes	*Staphylococcus Lactococcus Bacillus Brevibacillus*	3 2 5 1
	Actinobacteria	*Microbacterium Arthrobacter*	3 3
Endophyte: CMMF-EMS**	Firmicutes	*Bacillus*	5
	Proteobacteria (Gammaproteobacteria)	*Pantoea Enterobacter Serratia*	2 1 1
	Proteobacteria (Alphaproteobacteria)	*Ochrobactrum*	1
Rizhosphere: CMMF-EMS**	Firmicutes	*Bacillus*	2
Total			69

*Applied Microbiology Laboratory of Instituto de Ciências Biológicas of Universidade Federal de Minas Gerais. **Multifunctional Microorganisms Collection of EMBRAPA Maize and Sorghum.

### Quantitative Estimation of Inorganic Phosphate Solubilization by Bacteria in the Sessile and Planktonic States

**FIGURE 1:**
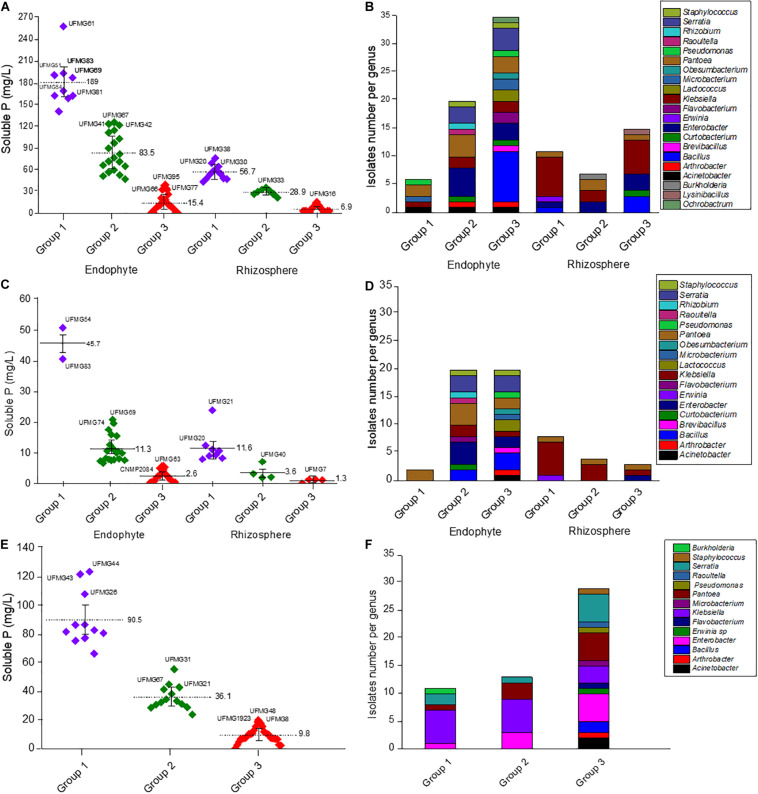
P solubilization from Araxá Phosphate (AP) **(A)**, and Iron Phosphate (Fe–P) **(C)** by planktonic cells and from AP by sessile cells **(E)** of the endophyte and rhizosphere bacteria. The bacteria were clustered into three groups according to their P solubilization efficiency, as follows: Group 1- high P solubilization; 2- medium P solubilization; and 3- low P solubilization. The average soluble *P*-values recorded as well as the best solubilizer in each group are indicated. The graphs to the right show the endophyte and rhizosphere bacterial genera distribution in each group in assays with AP **(B)** and Fe–P **(D)** under agitation and AP with sessile cells **(F)**. The identification of other isolates of each group obtained by K-means analysis is shown in the Supplementary Tables S2–S4.

We explored the potential of 101 endophyte and rhizosphere isolates (Table 2) to solubilize two different inorganic P sources, namely AP and Fe–P, using an *in vitro* quantitative approach (Figure 1). The highest values of soluble P from AP were observed in tests with the endophytic bacteria (*p* < 0.05), which ranged from 0 to 258 mg/L, when compared to the rhizospheric ones that ranged from 0 to 76.1 mg/L (Figure 1A). The soluble phosphorus variation factor was used to group the isolates according to their P- solubilization levels by the K-means method; then, the genus distribution in each group was made (Figures 1B,D,F and Supplementary Tables S2–S4). The endophytic bacteria cultivated with AP under agitation (Supplementary Table S2) were classified as follows: Group 1, with 10 isolates, P-solubilization varying from 140.7 to 258 and average of 189 mg/L; Group 2, with 19 isolates, P-solubilization varying from 47 to 125.7 and average of 83.5 mg/L; and Group 3, with 38 isolates, showing P-solubilization lower than 39 mg/L and an average value of 13 mg/L. The group with the best solubilizers included *Serratia* (3 isolates), *Pantoea* (2 isolates), *Klebsiella* (1 isolate), *Pseudomonas* (1 isolate), *Raoutella* (1 isolate), *Microbacterium* (1 isolate) and *Acinetobacter* (1 isolate) The best endophytic P-solubilizer was *Microbacterium* sp. UFMG61. Rhizosphere bacteria were classified as follows: Group 1, with 11 isolates and P solubilization varying from 43.7 to 76.1 mg/L and average of 56.7 mg/L; Group 2, with 7 isolates, P solubilization values varying from 21.1 to 35.5 mg/L and average of 28.9 mg/L; Group 3, with 17 isolates, P solubilization lower than 15.9 mg/L and average corresponding to 6.5 mg/L (Figure 1A). The group of best solubilizers also included the genera *Pantoea* (1 isolate) and *Klebsiella* (7 isolates), besides isolates of *Enterobacter* (1 isolate), *Bacillus* (1 isolate) and *Erwinia* (1 isolate). The best of all were *Pantoea* sp. UFMG38 and *Klebsiella* UFMG20, with P solubilization values of 76.1 and 68.9 mg/L, respectively.

**FIGURE 2:**
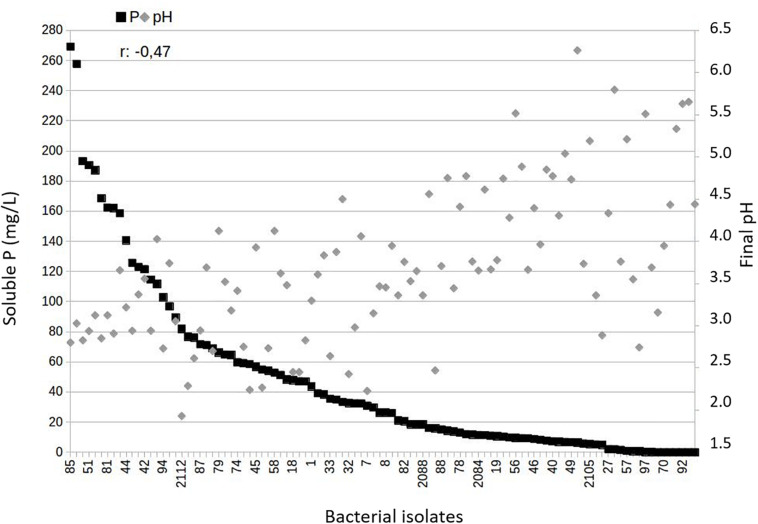
Relationship between soluble *P*-values and final pH in the assays of Araxá Phosphate solubilization under agitation for the endophyte and rhizosphere bacteria studied. The data show negative and significant correlation based on Pearson’s correlation coefficient for the endophytic and rhizospheric bacteria belonging to Group 1 (high solubilization) (*r*: –0.47; *p* = 6.2 × 10^–7^).

Because acidification is a major mechanism involved in P-solubilization, we also monitored pH variation and found lower values of pH in the culture media from treatments with endophyte and rhizospheric bacteria belonging to group 1 (Figure 2). The variables P solubilization and pH showed a negative correlation (*r*: −0.47, *p* = 6.2 × 10^−7^), as expected.

The amount of P solubilized from FeP in the solubilization tests were generally lower than those released from AP (Figure 1C). The clustering profile also changed, mainly regarding the components of group 1 of endophytic and rhizospheric isolates. The group 1 of endophytic bacteria is composed of two *Pantoea* isolates, UFMG54 and UFMG83, which were associated with the release of 51 mg/L and 41 mg/L of P, respectively. The 24 isolates of the group 2 promoted the release of 6.6 to 20.9 mg/L (average 11.34 mg/L) of P; and the 20 isolates in the group 3 promoted the release of P amounts lower than 5.8 mg/L from Fe–P, with an average of 1.14 mg/L. The distribution of bacterial genera per group is summarized in Supplementary Table S3.

As for the rhizospheric bacteria, an effect of the type of soil used in enrichment on the solubilization profile of Fe–P was observed (*p* < 0.05), and the isolates obtained from the enrichment with maize rhizospheric soil cultured with AP were more efficient than those obtained from the rhizosphere soil without the addition of P (*p* < 0.05). Group 1 included 8 isolates, identified as *Pantoea* sp. (1 isolate), *Klebsiella* sp. (6 isolates) and *Erwinia* sp. (1 isolate), which were associated with the release of 7.9 to 11.1 mg/L P, and an average of 11.57 mg/L P. Among them, six strains, namely UFMG14, UFMG16, UFMG20, UFMG21, UFMG29, and UFMG38, were isolated from the soil with AP. Group 2 includes 4 isolates able to promote the release of 1.9 mg/L to 7.1 mg/L P, with an average of 3.57 mg/L P; while group 3 has 3 isolates for which the measured P solubilization activity was lower than 1.4 mg/L P. In this assay, 20 isolates did not present Fe–P solubilization activity. The distribution of bacterial genera in each of these groups is summarized in Supplementary Table S4.

In the solubilization tests using Fe–P for both endophytic and rhizospheric bacteria, the pH values of the medium showed a very low correlation with the soluble P released in the medium, although there was significance (*r*: −0.25, *p* = 0.04, data not shown).

A group formed by 56 bacteria that stood out in the tests using agitation (i.e., planktonic cells) were evaluated for their AP solubilization ability in a static condition (i.e., sessile cells) (Figures 1E,F and Supplementary Table S3). As done previously, the bacteria were grouped based on their solubilization ability, as measured by the amount of phosphorus released in the medium. Group 1 (11 isolates) showed the highest levels of solubilization, with an average of 90.5 mg L^–1^ of P released in the medium. The group with an intermediate profile (group 2) released an average of 36.1 mg/L of P, while group 3, the one with the lowest potential for P-solubilization, showed an average of 8.3 mg/L of P released. Two endophytic strains of *Serratia marcescens* (UFMG44 and UFMG43) showed the highest P solubilization performance- (*p* < 0.05), with 124 and 122 mg/L of P released, respectively (Figure 1E).

### Influence of Bacterial Inoculation on the Growth of Millet Cultivated in Soils With Different Phosphate Sources

Six of the 101 bacteria showing P-solubilization ability *in vitro* were selected for testing in greenhouse conditions, to evaluate whether they can increase the millet growth properties when cultivated in soil added with cRP or TSP (Table 3). Overall, the averages of plant growth parameters were higher in soil added of P soluble source (TSP) than in the soil with cRP. However, the positive responses of bacterial inoculation on parameters of millet growth (foliar area, plant height, root and shoot dry weight, plant dry weight) and P absorption (root P, shoot P and pant P) was more frequent in the seedlings that grew in soil fertilized with cRP than in those that grew in soil fertilized with TSP.

**TABLE 3:** Effect of bacterial inoculations on level of phosphorus in the soil and physiological traits of millet seedlings cultivated in soil fertilized with triple superphosphate (TSP) or commercial rock phosphate (cRP) in greenhouse conditions.

Treatment	Soil P (mg/Kg)	Foliar area (cm^2^)	Plant height (cm)	Root dry weight (g per pot)	Shoot dry weight (g per pot)	Plant dry weight (g per pot)	Root P (g per pot)	Shoot P (g per pot)	Plant P (g per pot)
**Soil added with TSP**									
NI	6.4 ± 0.6^*a*^	549.5 ± 54.9^*a*^	140.0 ± 2.5^*a*^	5.2 ± 0.6^*a*^	37.8 ± 1.3^*b*^	43.0 ± 1.3^*a*^	3.7 ± 0.5^*a*^	35.2 ± 4.5^*a*^	38.9 ± 4.5^*a*^
*Bacillus megaterium* UFMG50	5.8 ± 2.0^*a*^	579.2 ± 34.6^*a*^	149.8 ± 2.9^*a*^	5.9 ± 0.8^*a*^	35.7 ± 2.8^*b*^	41.6 ± 2.9^*a*^	4.5 ± 1.1^*a*^	33.3 ± 0.4^*a*^	37.8 ± 1.4^*a*^
*Klebsiella variicola* UFMG51	6.3 ± 2.7^*a*^	634.2 ± 38.0^*a*^	145.3 ± 2.5^*a*^	6.7 ± 0.5^*b*^	36.2 ± 2.2^*b*^	42.9 ± 1.8^*a*^	5.6 ± 0.75^*b*^	39.2 ± 2.8^*b*^	44.7 ± 3.0^*b*^
*Pantoea ananatis* UFMG54	7.5 ± 3.9^*a*^	814.8 ± 93.7^*b*^	146.7 ± 3.0^*a*^	5.3 ± 0.2^*a*^	36.2 ± 1.4^*b*^	41.4 ± 1.2^*a*^	3.9 ± 0.3^*a*^	39.5 ± 3.9^*b*^	43.4 ± 3.9^*b*^
*Microbacterium* sp. UFMG61	5.7 ± 1.3^a^	656.0 ± 44.8^*a*^	143.5 ± 1.2^*a*^	5.4 ± 0.3^*a*^	37.2 ± 1.8^*b*^	42.6 ± 1.8^*a*^	4.4 ± 0.7^*a*^	42.5 ± 7.3^*b*^	46.9 ± 7.9^*b*^
*Pseudomonas* sp. UFMG81	6.0 ± 1.3^*a*^	597.1 ± 62.8^*a*^	141.2 ± 10.8^*a*^	6.6 ± 0.3^*b*^	32.8 ± 1.4^*a*^	39.4 ± 1,5^*a*^	5.3 ± 0.3^*b*^	33.9 ± 3.9^*a*^	39.2 ± 3.8^*a*^
*O. pseudogrignonense* CNPMS2088	7.5 ± 2.1^*a*^	634.4 ± 53.2^*a*^	147.3 ± 3.1^*a*^	5.8 ± 0.7^*a*^	37.2 ± 2.3^*b*^	43.0 ± 3.0^*a*^	4.5 ± 0.4^*a*^	34.9 ± 3.9^*a*^	39.4 ± 4.3^*a*^
**Soil added with cRP**									
NI	44.7 ± 3.3^*a*^	258.3 ± 36.4^*b*^	99.2 ± 2.0^*b*^	1.9 ± 0.2^*a*^	7.9 ± 0.7^*a*^	9.8 ± 0.8^*a*^	2.2 ± 0.2^*a*^	11.4 ± 0.7^*b*^	13.6 ± 0.7^*a*^
*Bacillus megaterium* UFMG50	64.4 ± 6.9^*c*^	300.8 ± 34.0^*c*^	109.0 ± 2.2^*c*^	2.8 ± 0.3^*b*^	9.2 ± 0.5^*b*^	11.9 ± 0.7^*b*^	3.4 ± 0.6^*b*^	13.1 ± 0.9^*c*^	16.5 ± 1.3^*b*^
*Klebsiella variicola* UFMG51	42.2 ± 4.8^*a*^	208.6 ± 40.8^*a*^	86.5 ± 5.0^*a*^	2.0 ± 0.4^*a*^	7.9 ± 0.5^*a*^	9.9 ± 0.8^*a*^	2.4 ± 0.4^*a*^	11.3 ± 0.8^*b*^	13.7 ± 1.0^*a*^
*Pantoea ananatis* UFMG54	55.1 ± 7.6^*b*^	218.9 ± 9.5^*a*^	92.0 ± 2.2^*a*^	2.0 ± 0.2^*a*^	7.6 ± 0.3^*a*^	9.6 ± 0.5^*a*^	2.3 ± 0.2^*a*^	11.6 ± 0.8^*b*^	13.9 ± 0.9^*a*^
*Microbacterium* sp. UFMG61	48.1 ± 6.1^*a*^	253.6 ± 8.5^*b*^	97.8 ± 3.6^*b*^	2.1 ± 0.1^*a*^	7.8 ± 0.5^*a*^	9.9 ± 0.6^*a*^	2.6 ± 0.2^*a*^	11.4 ± 0.5^*b*^	14.0 ± 0.5^*a*^
*Pseudomonas* sp. UFMG81	53.1 ± 7.2^*b*^	327.3 ± 45.6^*c*^	92.0 ± 2.2^*b*^	1.9 ± 0.1^*a*^	7.3 ± 0.2^*a*^	9.2 ± 0.2^*a*^	2.3 ± 0.1^*a*^	9.8 ± 0.4^*a*^	12.1 ± 0.4^*a*^
*O. pseudogrignonense* CNPMS2088	54.7 ± 4.6^*b*^	221.2 ± 27.2^*a*^	105.8 ± 6.8^*c*^	2.5 ± 0.7^*b*^	10.3 ± 1.18^*c*^	12.8 ± 1.8^*b*^	2.6 ± 0.5^*a*^	13.6 ± 1.8^*c*^	16.2 ± 2.3^*b*^
Negative control	1.8 ± 0.3	32.4 ± 2.8	42.8 ± 3.3	0.7 ± 0.2	1.0 ± 0.2	1.7 ± 0.3	0.73 ± 0.2	0.82 ± 0.2	1.56 ± 0.3

*Negative control, no inoculation and no P fertilization; NI, no inoculation. Data are the mean value of four replicates followed by standard deviation. The inoculation consisted in the addition of 10 mL/pot of the bacterial suspension, adjusted to an O.D_600_ of 1.0 at the time of planting. All traits were measured after 45 days of cultivation. Means followed by the same letter within the same column do not differ significantly from one another by the Scott-Knott test (*p*-value ≤ 0.05).*

In treatments with cRP, *B. megaterium* UFMG50 and *O. pseudogrignonense* CNPMS2088 significantly (*P* < 0.05) outperformed the other treatments, inoculated and not inoculated, in at least six physiological traits of millet or P content in the soil. UFMG50 showed the best performance, increasing all the parameters evaluated when compared to the uninoculated treatment: increasing foliar area (16.5%), plant height (9.9%) and root (47.45), shoot (16.5%) and plant biomass (21.4%), and P content in the shoot (14.9%), root (54.6%) and plant (21.3%), while *O. pseudogrignonense* CNPMS2088 promoted greater increase in plant height (6.7%), root (31.6%), shoot (30.3%) and plant biomass (30.6%) and P in the shoot (19.3%) and plant (19.1%). Strains UFMG50 and CNPMS2088, together with *Pantoea ananatis* UFMG54 and *Pseudomonas* sp. UFMG81, significantly increased soil P content in nearly 44.1, 23.3, 22.4, and 18.8%, respectively, when compared with soil without inoculation. Finally, the inoculation of seedlings with UFMG81 led to an increase in plant height and foliar area, reaching 92 cm and 327.3 g/pot.

In the treatment with TSP, *K. variicola* UFMG51 had the best performance, significantly increasing the root biomass in 28.9% and P content in the shoot, root and plant in 51.4, 11.4, and 14.9, respectively (*p* < 0.05), when compared to the non-inoculated control. UFMG54 inoculation promoted an increment of P in shoot and plant to values similar to UFMG 51, besides promoting an increase in foliar area (48.3%) in relation to non-inoculated pots. For these parameters, UFMG 81 promoted gains in root dry weight and root P content of 20.7 and 20.6%, respectively. However, no single isolate managed to significantly increase (*p* < 0.05) plant height, shoot and plant dry weight and soil P content simultaneously.

### Genome Analysis of Selected Bacteria

After confirming the ability of the six selected bacteria to improve P-solubilization *in vitro* and/or *in vivo*, we proceeded to sequencing and assembling their genomes. Though none of the genomes was closed (the number of contigs was between 22 and 73), we checked the assemblies for the presence of single copy orthologs (BUSCOs) at the level of order or class. The number of BUSCOs searched ranged between 292 and 782, depending on the taxon, and between 98 and 100% of the searched BUSCOs were found in all genomes only once, indicating the assemblies were of high quality.

For an improved taxonomic assignment of the six bacteria studied under greenhouse conditions we performed a genome-scale phylogeny and taxonomy analysis using the Type (Strain) Genome Server – TYGS (Meier-Kolthoff and Göker, 2019), and a MALDI-TOF mass spectrometry analysis based on the ribosomal protein profile (Table 4). The spectrometric analysis allowed the genus-level identification of four bacteria, UFMG50 (*Bacillus* sp.), UFMG51 (*Klebsiella* sp.), UFMG54 (*Pantoea* sp.) and UFMG81 (*Pseudomonas* sp.) since the scores for these bacteria were equal to or greater than 2.0 (Table 4). UFMG61 was identified as probable genus (*Microbacterium* sp.) according to the MALDI-TOF score, but no correspondence at the genus level was found for CNPMS2088 in the MALDI-TOF spectral library (Table 4). UFMG50, UFMG51, UFMG54 and CNPMS2088 were identified by TYGS as *B. megaterium*, *K. variicola*, *P. ananatis*, and *O. pseudogrignonense*, respectively (Table 4).

**TABLE 4:** Taxonomy of the six bacteria selected in the *in vitro* tests according to the Type (Strain) Genome Server (TYGS) and the protein profile analysis using matrix-assisted desorption ionization flight time mass spectrometry (MALDI-TOF/MS).

Strain	TYGS analysis		MALDI-TOF/MS Score
	Taxon	d0 – d4 -d6 (%)*	
UFMG50	*Bacillus megaterium*	71.8 – 72.1 – 74.3	*Bacillus* sp. /2.1 (secure genus identification)
UFMG51	*Klebsiella variicola*	82.4 – 92.7 – 87.0	*Klebsiella* sp. / 2.3 (secure genus identification)
UFMG54	*Pantoea ananatis*	86.6 – 91.9 – 90.2	*Pantoea* sp. / 2.0 (secure genus identification)
UFMG61	*Microbacterium* sp.	47.8 – 26.8 – 41.2	*Microbacterium* sp. / 1.9 (probable genus identification)
UFMG81	*Pseudomonas* sp.	61.2 – 38.2 – 56.0	*Pseudomonas* sp. / 2.0 (secure genus identification)
CNPMS2088	*Ochrobactrum pseudogrignonense*	71.8 – 71.4 – 74.2	Not detected

*Digital DNA-DNA hybridization (dDDH) values (Meier-Kolthoff and Göker, 2019): d_0_ (GGDC formula 1): length of all HSPs divided by total genome length. d_4_ (GGDC formula 2): sum of all identities found in HSPs divided by overall HSP length. d_6_ (GGDC formula 3): sum of all identities found in HSPs divided by total genome length.

**FIGURE 3:**
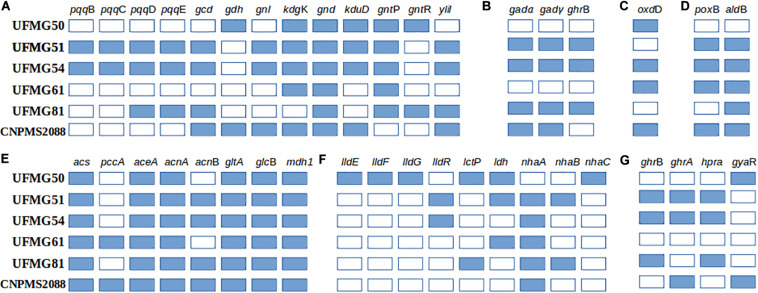
Presence or absence of genes related to the synthesis of organic acids: **(A)** Gluconic, **(B)** ketogluconic, **(C)** Formic, **(D)** Acetic, **(E)** Glyoxylic, **(F)** Lactic, and **(G)** Glycolic in the six bacterial genomes (*B. megaterium* UFMG50, *K. variicola* UFMG51, *P. ananati*s UFMG54, *Microbacterium* sp. UFMG61, *Pseudomonas* sp. UFMG81 and *O. pseudogrignonense* CNPMS2088). Pyrroloquinoline quinone biosynthesis protein B (*pqq*B), pyrroloquinoline-quinone synthase (*pqq*C), pyrroloquinoline quinone biosynthesis protein D (*pqq*D), PqqA peptide cyclase (*pqq*E), quinoprotein glucose dehydrogenase (*gcd*), glucose 1-dehydrogenase (*gd*h), gluconolactonase (*gn*l), 2-dehydro-3-deoxygluconokinase (*kdg*K), 6-phosphogluconate dehydrogenase (*gnd*), 2-dehydro-3-deoxy-D-gluconate 5-dehydrogenase (*kdu*D), gluconate:H+ symporter (*gnt*P), gluconate operon transcriptional repressor (*gnt*R), aldose sugar dehydrogenase (*yliI*), gluconate 2-dehydrogenase alpha chain (*gad*α), gluconate 2-dehydrogenase gamma chain (*ga*dγ), 2-keto-D-gluconate reductase-gluconate 2-dehydrogenase (*ghr*B), oxalate decarboxylase (o*xd*D), pyruvate dehydrogenase (*pox*B), aldehyde dehydrogenase (*ald*B), acetyl-CoA synthetase (*acs*), propionyl-CoA carboxylase alpha chain (*pcc*A), Isocitrate lyase (*ace*A), aconitate hydratase A (*acn*A), aconitate hydratase B (*acn*B), citrate synthase (*glt*A), malate synthase (*glc*B), malate dehydrogenase (*mdh*1), L-lactate dehydrogenase complex protein (*lld*E), L-lactate dehydrogenase complex protein (*lld*F), L-lactate dehydrogenase complex protein (*lld*G), L-lactate dehydrogenase operon regulator (*lld*R), lactate permease (*lct*P), L-lactate dehydrogenase (*ldh*), Na+:H+ antiporter NhaA family (*nha*A), Na+:H+ antiporter NhaB family (*nha*B), malate-2H(+)/Na(+)-lactate antiporter (*nha*C), glyoxylate/hydroxypyruvate/2-ketogluconate reductase (*ghr*B), glyoxylate/hydroxypyruvate reductase (*ghr*A), glycerate dehydrogenase (*hpra*), glyoxylate reductase (*gya*R). A list with KO and GOG numbers can be accessed in Supplementary Table S6.

Given their interesting properties, we checked these strains’ genomes for the presence of genes possibly involved in plant growth promotion, especially those related to P metabolism and to organic acids synthesis (Figure 3 and Supplementary Table S5). Importantly, we detected the full *pst* operon in the genomes of all strains, except for the gene *pst*A in the genome of *Microbacterium* sp. UFMG61. In addition, all strains have the gene *pit*A, which codes for the low affinity inorganic phosphate transporter, except *O. pseudogrignonense* CNPMS2088. We also searched for other components of the bacterial P signaling pathway, that includes the two-component regulatory proteins PhoB/PhoR, and the key negative regulator of phosphate transport, PhoU. All strains have a copy of the full Pho regulon, except strain UFMG61 (*Microbacterium* sp.), in which genome we did not find the two main regulatory components of the Pho system (*pho*B and *pho*R). In addition, the transcriptional regulatory protein PhoP seems to be especially important in strain UFMG50 (*B. megaterium*), as the *pho*P gene was found in multiple copies in the genome. On the other hand, the gene for the phosphate transport regulator PhoU is present in the genomes of all strains, except in that of strain UFMG50 (*B. megaterium*). For pathways related to the catabolism of phosphonates and phosphites, the *phn*GHIJK C-P lyase gene were found only in the genomes of strains UFMG51 (*K. variicola*), UFMG54 (*P. ananatis*), and *O. pseudogrignonense* CNPMS2088. The *phn*XW gene (phosphonatase) was found only in the genomes of strains UFMG50 (*B. megaterium*) and UFMG81 (*Pseudomonas* sp.). Genes coding for alkylphosphonate transport and utilization (PhnA phosphonoacetate hydrolase and PhnB permease) were found exclusively in the genomes of strains UFMG50 (*B. megaterium*) and UFMG51 (*K. variicola*). Moreover, genes for the synthesis of exopolyphosphatase (*ppx*) and polyphosphate kinase (*ppk*) are present in the genomes of all six strains selected in this study, except that *ppx* was not found in the genome of *O. pseudogrignonense* CNPMS2088. Except *Microbacterium* sp. UFMG51, all other strains genomes have an alkaline phosphatase gene; and UFMG50 has many copies of this gene and of its transcriptional regulator.

We also analyzed the gene content related to the synthesis of organic acids (Figure 3), which is a major mechanism of phosphate solubilization by bacteria. Several genes related to the metabolism of gluconic, ketogluconic, lactic, formic, acetic, glyoxylic, and glycolic acids were detected in all genomes (Figure 3). Strains UFMG51 (*K. variicola*) and UFMG54 (*P. ananatis*) have the highest diversity of genes, especially those for the synthesis of gluconic, acetic and glycolic acids (Figures 3A,D,G); CNPMS2088 (*O. pseudogrignonense*) has all genes necessary for the synthesis of glyoxylic and acetic acids, and some of these enzymes are also key to the production of succinic and citric acid. UFMG50 (*B. megaterium*) has the highest diversity of genes for the synthesis of lactic acid. The genome of strain UFMG54 (*P. ananatis*) has all genes detected for the synthesis of ketogluconic, formic and acetic acids.

## Discussion

### Enrichment of Bacterial Taxa From Soil Rhizospheric Cultivated With RP

In this study, we found changes in heterotrophic bacterial groups selected during acclimation in medium with AP promoted by enrichment technique using two sources of bacteria, rhizosphere soil of plants of maize cultivated with AP and maize rhizosphere soil without P (Table 2). In general, most bacteria isolated belong to the Gammaproteobacteria class and to the Enterobacteriaceae family. They were represented mainly by the genera *Klebsiella* sp. (62.5%) and *Pantoea* sp. (25%), when soil added with AP was used as inoculum for enrichment, and by *Enterobacter* sp. (50%) and *Klebsiella* sp. (25%) when soil without the addition of P was used. A similar profile, with the enrichment of taxa of Enterobacteriaceae family, was also observed in a previous study conducted by our group using metataxonomic approach (Silva et al., 2017). The Enterobacteriaceae are ubiquitously distributed, being reported in diverse environments, such as soils, water, plants and clinical samples (Podschun et al., 2001; Abreu et al., 2017; Silva et al., 2017). Members of this family are described as inorganic P solubilizers and/or adapted to environments with low levels of soluble phosphorus (Silva et al., 2017), as in the selective medium used for the enrichment and isolation of these bacteria (Ikeda et al., 2013).

### Bacterial Potential of P Solubilization Depends on the Source, Strain and Availability of Oxygen in the Medium

In the assays to evaluate the P-solubilization capacity of bacteria, we used the Fe–P, because P is typically associated with Fe in most tropical soils, and Araxá Phosphate (AP), as P–Ca source of low water solubility. The evaluated bacteria performed better on the AP source, as evidenced by higher amount of P detected in the solubilization tests when compared to that measured in tests using FeP (Figure 1). In fact, calcium-bound phosphate, as in the case of AP, has greater solubility than phosphate bound to Fe or Al (Viégas et al., 2010). This pattern of greater solubilization of calcium phosphate mediated by microorganisms in *vitro* assays has been observed previously (Silva et al., 2014; Spagnoletti et al., 2017; Matter et al., 2020).

*Microbacterium* sp. UFMG61 was the bacterium that best solubilized AP (*p* < 0.05), reaching 258 mg/L of P after 48 h (Figure 1A). These results are promising when we consider previous study by our research group (Gomes et al., 2014) in which the incubation period of AP solubilization for bacteria was 10 days and reached a maximum of 100.7 mg/L, a value 2.5 times lower than that found in this study. Also, in the most recent study by Hongyang et al. (2020) lower values of P released were observed (90.2 mg/L) from the rock phosphate of Khouribga after 7 days of bacterial incubation. However, many factors may be acting for this better result of the group 1 – endophytes bacteria (Figure 1B), such as, for example, the ability to tolerate the impurities present in the rock phosphate that are also released during the P solubilization process. Mendes et al. (2013) observed that the fluoride release from AP reduced, in 55%, the P solubilization efficiency of *Aspergillus niger.*

Moreover, generally, lowering the pH correlates with increasing levels of solubilized P from calcium-bound phosphate, as observed for the best solubilizers of AP under study (Figure 2). The acidification of the medium may occur due the production of organic acids, H^+^ ions release during the assimilation of NH_4_^+^ or through other metabolic reactions that trigger the extrusion of protons, such as respiration, these are the main mechanisms reported as responsible for making P available from insoluble sources, such as rock phosphates (Illmer and Schinner, 1995). Furthermore, the efficiency of organic acids in the solubilization of P is related to the formation of complexes that act in the chelation of the ions that are P linked, then making the P soluble (Mendes et al., 2015). Another observation in this study is that the bacterial community showed a variable P solubilization pattern with genera, i.e., independent-genera, once that different isolates of the same genera were observed in the three groups generated by K-means analysis (Figure 1). There was also a greater potential for solubilization of AP by endophytic bacteria compared to rhizosphere bacteria (Figure 1A, *p* < 0.05). This effect of the origin site of bacteria of maize microbiome on P solubilization still needs to be further investigated, since numerous studies report this potential for bacteria isolated both from internal maize tissues (Matos et al., 2017; de Sousa et al., 2020) and the plant rhizosphere (Gomes et al., 2014; Li et al., 2017). It was expected that this greater potential was more related to rhizospheric bacteria that live in soil containing P insoluble forms. But, an aspect to be considered is that plant endosphere bacteria include facultative and obligatory endophytes. The facultative ones can have a stage outside the host plants (Hardoim et al., 2008) and originate from the soil. Therefore, the endophytic community likely represents a subset of the wider microbial population of the rhizosphere and it would reflect differences induced by agronomic practices (Miliute et al., 2015).

In the experiments in polystyrene plates (sessile cells), it was also observed high capacity of AP solubilization by the *S. marcescens* UFMG44 and *S. marcescens* UFMG43 reaching levels of 124 and 122 mg/L of P released, respectively (Figure 1E). These values are higher than those found in other studies under agitation conditions that also prospected bacteria through analysis of the capacity for P solubilization (Gomes et al., 2014; Motamedi et al., 2016). Probably, in this work, the experimental conditions produced anaerobic or microaerobic environments that led the bacteria to alter their metabolism and perform P solubilization, even in conditions of oxygen limitation. *S. marcescens* is a facultative anaerobe, being able to grow both in the presence and in the absence of oxygen, using for example the fermentative pathways (Jung et al., 2007).

### Bacterial Inoculation Has Major Benefit in the Millet-cRP Assays

From the analysis of the promotion of millet growth by the six selected isolates (Table 3) it was observed as expected, the results of millet growth are higher in the soil added with P soluble (TSP) than with cRP. However, when analyzing the effect of inoculation of each P source, the bacterial inoculation on millet growth was more beneficial to seedlings that grew in soil fertilized with cRP than to those that grew in soil fertilized with TSP. This suggests that combining cRP with P-solubilizing bacteria to increase the P supply to the plant is an efficient approach, especially in the management systems that recommend not using chemical fertilizers such as TSP.

In fact, cRP is a slow release fertilizer and this is an advantage feature that can minimize P loss, contributing to the better use of this important element in tropical soils. Previous studies by our group using field cultivation of maize plants in RP-fertilized soil for 3 years showed biomass production and grain yield comparable to that of the plants cultivated in the TSP-added soil (Silva et al., 2017). Based on metataxonomic data, the authors hypothesized that the long-term cRP-fertilization promoted a greater relative abundance of microbial taxa related to P solubilization/acquisition in this soil and consequently helped its release and availability to plants.

A similar process may have occurred in the experiment with millet, where the P levels in the soil at the end of the experiment were higher in treatments with cRP than TSP, and this content of P in the soil was further increased with the inoculation of some of the bacteria in this study (i.e., *B. megaterium* UFMG50, *P. ananatis* UFMG54, *Pseudomonas* sp. UFMG81 and *O. pseudogrignonense* CNPMS2088). The largest increments were observed with *B. megaterium* UFMG50, which also improved all growth parameters of millet cultivated with cRP compared to those cultivated in uninoculated soil (Table 3). This may be a strong indication that this increase in P occurred through processes carried out by this strain, resulting in more P available to support plant growth. The potential of *B. megaterium* in releasing P from RP is well known and has been correlated with the production of organic acids (Do Carmo et al., 2019). A positive effect of the inoculation on the growth parameters of plants cultured in soil fertilized with RP was reported in other studies (Ribeiro et al., 2018; Hasan et al., 2019). Another important characteristic of the *Bacillus* genus is their high potential for resistance to a range of stress conditions in the soil due to their ability to form spores. This characteristic contributes to the good performance of *B. megaterium* and to its survival in soil, and consequently increases the chance that this bacterium promotes plant growth (Nascimento et al., 2020).

*O. pseudogrignonense* produced the largest increase in P content in soil and in all parameters related to millet growth promotion, but leaf area. This bacterium seems to be an important biostimulant of plant growth, as previously reported by Mishra et al. (2017). These authors reported an increase in the biomass of roots, aerial part and number of leaves of maize plants after treatment of maize seeds with an *Ochrobactrum* strain that was also tolerant to abiotic stresses, such as temperature, salinity and drought. Indeed, the plant growth promotion ability of *Ochrobactrum* spp. has been linked to ACC deaminase activity, nitrogen fixation, P solubilization, siderophore and indole acetic acid (IAA) production and biocontrol agent (Zhang et al., 2017; Singh et al., 2018; Navarro-Torre et al., 2019; Zang et al., 2019).

Although bacterial inoculation had an overall smaller effect on plant growth in soil added with TSP, when compared to other treatments, strains *K. variicola* UFMG51 and *P. ananatis* UFMG54 improved three and two parameters of millet growth, respectively (Table 3). The mechanisms used by *Klebsiella* spp. to promote plant growth include N_2_ fixation, ammonia production, phosphate solubilization and IAA production (Mazumdar et al., 2018). As TSP is a soluble P source, P solubilization is expected to occur readily, and this may have influenced bacterial growth. In the case of *P. ananatis*, it is interesting to observe the versatility of this species, since some strains have proven effective as plant-growth promoters (Da Silva et al., 2015), as we also found in this study.

Another interesting observation is that some bacteria perform best in promoting the growth of millet in soil added with cRP (*B. megaterium* UFMG50 and *O. pseudogrignonense* CNPMS2088), while no effect is observed in soil added with TSP. We believe cRP may be acting as a selective factor for the activity of some inoculated bacteria similarly to what we observed in a previous study (Silva et al., 2017).

### Genomic Features Support the Bacterial Potential for Rock Phosphate Solubilizing and Promotion Plant Growth

To verify whether the P solubilization ability observed *in vitro* and *in vivo* is supported by genetic characteristics, we sequenced the genome of the six bacteria evaluated for millet growth promotion under greenhouse conditions. While this does not allow us to infer strain efficiency, we observe that all strains have the genetic potential to synthesize at least one of the organic acids known to promote phosphate solubilization, such as gluconic, 2-keto gluconic, acetic, lactic, glyoxylic, glycolic and formic acids (Figure 3). These acids are produced either as metabolic products or as intermediates of the carbon metabolic pathways. Gluconic acid and 2-keto gluconic acid are synthesized by the peripheral oxidation of glucose by pyrroloquinoline quinone-linked glucose dehydrogenase (PQQ-GDH) and gluconate dehydrogenase (GADH), respectively (Ramachandran et al., 2006). Acetic acid can be produced from pyruvate in a single-step oxidation reaction catalyzed by enzyme pyruvate oxidase B (POXB) or in multistep reactions involving the pyruvate dehydrogenase complex (PDC) and acetyl-CoA. Lactic acid can be produced from pyruvate due the action of lactate dehydrogenase, while citrate and succinate are key intermediates of the citrate cycle. Glyoxylic acid is produced in the glyoxylate in a reaction catalyzed by isocitrate lyase (ICL), which can be transformed to oxalic acid by glyoxylate oxidase action or reduced to glycolate by glyoxylate, hydroxypyruvate or 2-ketogluconate reductases. Finally, formic acid can be produced from oxalic acid by the action of oxalate decarboxylase (*oxd*D). We observed that the profile of genes related to the synthesis of organic acids alone do not fully explain the potential for AP solubilization of some microorganisms, as observed for *Microbacterium* sp. UFMG61 (Figure 3 and Supplementary Table S2), which showed a high *in vitro* P solubilization ability together with a low diversity of genes for organic acids synthesis. We hypothesize that other characteristics of this strain may be contributing to its ability to solubilize P. For instance, proton extrusion mechanisms or the regulation of gene expression may influence the phenotype, making it difficult to make a correlation at the genomic level. On the other hand, UFMG51 (*K. variicola*), UFMG81 (*Pseudomonas sp.*) and UFMG54 (*P. ananatis*) showed a high potential for AP solubilization, and classified as the highest P solubilization efficiency group among the endophytic bacteria evaluated *in vitro*. These strains also showed the highest number and diversity of genes for the biosynthesis of the major organic acids investigated, except for lactic acid (Figure 3). These are strong indicators of the possible mechanisms related to medium acidification or ion chelation promoted by acids (Mendes et al., 2015) in the assays with these bacteria acting for the solubilization of P. Besides having genes for the synthesis of GDH and its cofactor (*pqq* – pqqBCDE operon), the strains have the *ylil* gene encoding a PQQ cofactor-dependent soluble aldose sugar dehydrogenase, which is able to oxidase glucose to gluconolactone with subsequent hydrolysis to gluconic acid, a function similar to that of the *gdh* gene product.

Interestingly, *B. megaterium* UFMG50, the strain with best results in the *in vivo* experiment of millet growth promotion in soil added with cRP has genes related to the production of gluconic, lactic and formic acids (Figure 3). OxdD, besides to contributing to P solubilization by catalyzing the formic acid synthesis, confers the ability to cope with the acidity conditions generated during the P solubilization process. This was verified in studies with *B. subtilis*, suggesting that this enzyme protects cells against low pH stress by consuming protons via oxalic acid decarboxylation (Tanner et al., 2001; MacLellan et al., 2009).

The production of phytohormones such as auxins (IAA) and cytokinins by bacteria is also another beneficial effect for plants (Marchant et al., 2002; Jang et al., 2015). Thus, looking for these genes in the genomes of the six bacteria evaluated with the millet experiment, we observed that *B. megaterium* UFMG50 stood out for presenting the largest number of genes related to production of IAA (five genes) and cytokinins (four genes) (unpublished data). Thus, we suggest that this bacterium was able to solubilize P from cRP in the rhizosphere of millet in a greenhouse, most likely through the release of organic acids, but may also have used other characteristics that favored the development of the plant such as stimulating the plant tissues through the production of IAA and cytokinins.

Regarding the high-affinity and high-velocity inorganic P acquisition system (PstSCAB), it was observed that all six genomes have genes for this system (Supplementary Table S5). The PstSCAB system has high phosphate specificity and is not sensitive to inhibition by phosphonates (Yuan et al., 2006). This may contribute to the adaptation of the bacterial bioinoculants under P deprivation conditions, such as those they may encounter when first inoculated in poor soils or in soils amended with less soluble P sources, such as RP. Moreover, all strains have a copy of the full Pho regulon, except strain UFMG61 (*Microbacterium* sp.), which seems to lack the two main regulatory components of the Pho system (*pho*B and *pho*R). The gene for the phosphate transport regulator PhoU is present in the genomes of all strains, except in that of strain UFMG50 (*B. megaterium*). Whether the absence of some genes represents an assembly artifact or a genuine biological event remains to be determined, but the confirmed presence of either *pho*U or *pho*BR has some important implications, as discussed by Vuppada et al. (2018). According to these authors, when P levels are low, PhoR phosphorylates the transcriptional regulator PhoB, which activates the expression of several genes involved in phosphate transport. On the other hand, when P levels are high, the PhoU protein modulates the Pst system transport rate. This rapid adaptation to high phosphate concentrations may be key to the survival of bioinoculant strains, as an excess Pi uptake could be toxic for the cell. Only one genome (*K. variicola* UFMG51) has a copy of a Pi-starvation response (PSR) gene, *psi*F, which is inducible under conditions of Pi starvation, and responsible for the activation of proteins that aid in the transport of inorganic phosphate, such as the phosphate (Pho) regulon and the phosphate-specific transporters (PstSCAB).

With regard to the catabolism of phosphonates and phosphites, the two strains that likely lack the Pho system (*Microbacterium* sp. UFMG61 and *O. pseudogrignonense* CNPMS2088) contain the full *phn*CDE operon (Supplementary Table S5), which was recently demonstrated to be a genuine phosphate transport system, as it supports growth with Pi in the absence of canonical Pi-transport systems (Stasi et al., 2019). Among the six strains, only two (*B. megaterium* UFMG50 and *Pseudomonas* sp. UFMG81) contain the genes *phn*W and *phn*X, which encode a transaminase and a phosphonatase, respectively. The *ptx and htx* operons, involved in the uptake and oxidation of the inorganic reduced phosphorus (P) compounds phosphite and hypophosphite, respectively, were not located in any of the genomes of the strains we studied. However, the genomes of all strains, except that of *Microbacterium* sp. UFMG61, do contain the gene *pho*A (alkaline phosphatase), which was reported to be involved in the oxidation of phosphites to P (Achary et al., 2017). Another interesting feature in the studied genomes is the ability to synthesize and mobilize inorganic polyphosphates (polyP). These molecules are known to fulfill a number of different functions in bacteria, such as resistance to stress, biofilm formation, quorum sensing and virulence (Xie and Jakob, 2019). In addition, as discussed by Albi and Serrano (2016) polyP also serve as a reservoir of inorganic P that can be mobilized when needed. This is a desirable genetic feature in bacterial bioinoculants, and genes for the synthesis of exopolyphosphatase (*ppx*) and polyphosphate kinase (*ppk*) are present in the genomes of all six strains selected in this study, except *ppx*, which is missing in the genome of *O. pseudogrignonense* CNPMS2088.

## Conclusion

In this study, we found that the bacterial efficiency for P solubilization varies widely at strain level. By screening 101 different bacteria, it was possible to observe that the site of origin can influence the phenotypic traits of isolates. Endophytic bacteria were more efficient in AP solubilization than bacteria isolated from rhizospheric soil. We observed that treatments based in a combination of bacterial inoculation and cRP-fertilization promoted millet growth to certain levels, though lower than those observed for TSP fertilization alone. Together with that, we observed that several isolates increased P both in soil and in plant tissues, suggesting that the increase in residual P in soil over time can supply the demand for this nutrient by plants. Of the six bacteria selected from P solubilization tests *in vitro*, all contributed to the improvement of at least one of the parameters of millet growth, indicating that the method used in this work was suitable for the selection of good candidates for bioinoculants for plant growth promotion. Furthermore, according to the analysis and detection of PCP genes in the studied genomes, we suggest that the production of organic acids and production of phytohormones are among the mechanisms that contribute to the promotion of millet growth. Hence, we propose that RP and the isolates described herein are used as adjuvants to a P-fertilization strategy in tropical soils. This strategy is important to alleviate the harmful effects of chemical P fertilizers by gradually solubilizing less soluble forms of P, such as RP, and making them available in soil for plant uptake.

## Data Availability Statement

The datasets presented in this study can be found in online repositories. The names of the repository/repositories and accession number(s) can be found in the article/Supplementary Material.

## Author Contributions

VLD and CAO conceived and supervised the study and acquired funding. UCS, SC-O, and LRL performed the computational analyses. UCS, LFF-J, ACF, and DRCS performed the microbiological and biochemical analyses. UCS performed the greenhouse experiments. VLD, UCS, and SC-O critically analyzed the results and wrote the manuscript. All the authors have read and approved the manuscript.

## Conflict of Interest

The authors declare that the research was conducted in the absence of any commercial or financial relationships that could be construed as a potential conflict of interest.

## Footnotes

1 http://asparagin.cenargen.embrapa.br/phph/

2 https://tygs.dsmz.de
