# Supplementary material for: Genomic and Phenotypic Insights Into the Potential of Rock Phosphate Solubilizing Bacteria to Promote Millet Growth in vivo
Source: Front Microbiol. 2021 Jan 7;11:574550. doi: 10.3389/fmicb.2020.574550 (PMC7817697; doi:10.3389/fmicb.2020.574550)
Supplement: Supplementary file 1 [file Table_1.pdf]

**Table S1-** Description of the bacteria used in this study regarding taxonomic identity and isolation site.

| Source | Bacterial isolate | Isolation site |
|---|---|---|
| LMA-UFMG | *Enterobacter* sp. UFMG45 | Endophyte/Leaf |
| | *Serratia marcescens* UFMG48 | |
| | *Klebsiella* sp. UFMG51 | |
| | *Pantoea ananatis* UFMG54 | |
| | *Pantoea* sp. UFMG55 | |
| | *Microbacterium* sp. UFMG56 | |
| | *Enterobacter* sp. UFMG58 | |
| | *Pantoea* sp. UFMG59 | |
| | *Obesumbacterium proteus* UFMG60 | |
| | *Enterobacter* sp. UFMG65 | |
| | *Lactococcus lactis* UFMG66 | |
| | *Pantoea vagans* UFMG67 | |
| | *Klebsiella* sp. UFMG79 | |
| | *Staphylococcus* sp. UFMG90 | |
| | *Curtobacterium flaccumfaciens* UFMG91 | |
| | *Staphylococcus epidermidis* UFMG92 | |
| | *Pantoea* sp. UFMG93 | |
| | *Serratia marcescens* UFMG41 | Endophyte/Root |
| | *Serratia marcescens* UFMG43 | |
| | *Serratia marcescens* UFMG44 | |
| | *Bacillus* sp. UFMG46 | |
| | *Bacillus* sp. UFMG47 | |
| | *Bacillus* sp. UFMG49 | |
| | *Bacillus megaterium* UFMG50 | |
| | *Bacillus* sp. UFMG52 | |
| | *Acinetobacter* sp. UFMG62 | |
| | *Arthrobacter ureafaciens* UFMG63 | |
| | *Arthrobacter* sp. UFMG64 | |
| | *Acinetobacter* sp. UFMG68 | |
| | *Rhizobium pusense* UFMG71 | |
| | *Enterobacter* sp. UFMG72 | |
| | *Acinetobacter* sp. UFMG73 | |
| | *Pantoea* sp. UFMG74 | |
| | *Enterobacter* sp. UFMG75 | |
| | *Lactococcus* sp. UFMG76 | |
| | *Staphylococcus* sp. UFMG77 | |
| | *Pseudomonas* sp. UFMG78 | |
| | *Acinetobacter* sp. UFMG80 | |
| | *Klebsiella* sp. UFMG82 | |
| | *Pantoea* sp. UFMG83 | |
| | *Enterobacter* sp. UFMG84 | |
| | *Serratia marcescens* UFMG85 | |
| | *Enterobacter* sp. UFMG86 | |
| | *Flavobacterium acidificum* UFMG88 | |
| | *Flavobacterium acidificum* UFMG89 | |
| | *Serratia* sp. UFMG94 | |
| | *Klebsiella* sp. UFMG95 | |
| | *Arthrobacter* sp. UFMG96 | |
| | *Curtobacterium albidum* UFMG97 | |
| | *Microbacterium testaceum* UFMG53 | Endophyte/Sap |
| | *Brevibacillus* sp. UFMG57 | |

| | | |
|---|---|---|
| | *Microbacterium* sp. UFMG61 | |
| | *Raoultella* sp. UFMG69 | |
| | *Klebsiella* sp. UFMG70 | |
| | *Pseudomonas putida* UFMG81 | |
| | *Klebsiella* sp. UFMG87 | |
| CMMF-EMS | *Pantoea ananatis* CNPMS1934 | Endophyte/Leaf |
| | *Enterobacter asburiae* CNPMS2084 | |
| | *Pantoea* sp. CNPMS2105 | |
| | *Bacillus* sp. CNPMS2110 | |
| | *Ochrobactrum* sp. CNPMS2088 | Endophyte/Root |
| | *Bacillus* sp. CNPMS2106 | |
| | *Bacillus* sp. CNPMS2111 | |
| | *Serratia marcescens CNPMS2112* | |
| CMMF-EMS | *Bacillus* sp. CNPMS116<br>*Bacillus* sp. CNPMS119 | Rhizosphere soil of maize plant |
| LMA-UFMG | *Klebsiella* sp. UFMG1 | Rhizosphere soil of maize plants cultivated without P fertilization (This study) |
| | *Klebsiella* sp. UFMG2 | |
| | *Erwinia* sp. UFMG4 | |
| | *Enterobacter* sp. UFMG5 | |
| | *Klebsiella* sp. UFMG6 | |
| | *Pantoea* sp. UFMG7 | |
| | *Enterobacter* sp. UFMG8 | |
| | *Enterobacter* sp. UFMG9 | |
| | *Enterobacter* sp. UFMG11 | |
| | *Burkholderia* sp. UFMG26 | |
| | *Curtobacterium* sp. UFMG27 | |
| | *Enterobacter* sp. UFMG28 | |
| | *Enterobacter* sp. UFMG30 | |
| | *Enterobacter* sp. UFMG31 | |
| | *Klebsiella* sp. UFMG33 | |
| | *Klebsiella* sp. UFMG35 | |
| | *Pantoea* sp. UFMG36 | Rhizosphere soil of maize plants cultivated with AP (This study) |
| | *Pantoea* sp. UFMG38 | |
| | *Klebsiella* sp. UFMG39 | |
| | *Pantoea* sp. UFMG40 | |
| | *Bacillus* sp. UFMG13 | |
| | *Klebsiella* sp. UFMG14 | |
| | *Klebsiella* sp. UFMG16 | |
| | *Enterobacter* sp. UFMG18 | |
| | *Klebsiella* sp. UFMG19 | |
| | *Klebsiella* sp. UFMG20 | |
| | *Klebsiella* sp. UFMG21 | |
| | *Klebsiella* sp. UFMG23 | |
| | *Lysinibacillus* sp. UFMG25 | |
| | *Klebsiella* sp. UFMG29 | |
| | *Klebsiella* sp. UFMG32 | |
| | *Bacillus* sp. UFMG34 | |
