# Supplementary material for: Genomic and Phenotypic Insights Into the Potential of Rock Phosphate Solubilizing Bacteria to Promote Millet Growth in vivo
Source: Front Microbiol. 2021 Jan 7;11:574550. doi: 10.3389/fmicb.2020.574550 (PMC7817697; doi:10.3389/fmicb.2020.574550)
Supplement: Supplementary file 2 [file Table_2.pdf]

Table S2 – Clustering of bacteria based on the profile of Araxá phosphate (AP) solubilization in assays under agitation according the K-means analysis.

Bacterial genera	Group 1 (high solubilization)		Group 2 (medium solubilization)		Group 3 (low solubilization)	
	ENDO	RIZO	ENDO	RIZO	ENDO	RIZO
Acinetobacter	UFMG62		UFMG68		UFMG80	
Arthrobacter			UFMG96		UFMG64	
Bacillus		UFMG34			UFMG46, UFMG47 UFMG49, UFMG50 UFMG52, UFMG1920 UFMG1923, UFMG2106, CNPMS 2111	UFMG13, CNPMS 116 CNPMS 119
Brevibacillus					UFMG57	
Burkholderia				UFMG26		
Curtobacterium			UFMG91		UFMG97	UFMG27
Enterobacter		UFMG18 UFMG30	UFMG45, UFMG58 UFMG65 UFMG75 UFMG84	UFMG31 UFMG8	UFMG72, UFMG86 CNPMS 2084	UFMG5 UFMG11, UFMG28
Erwinia		UFMG04				
Flavobacterium					UFMG88 UFMG89	
Klebsiella	UFMG51	UFMG01, UFMG02 UFMG06, UFMG14 UFMG20, UFMG21	UFMG79, UFMG87	UFMG32 UFMG33	UFMG82, UFMG95	UFMG16, UFMG19 UFMG23, UFMG29 UFMG35, UFMG39
Lactococcus					UFMG66 UFMG76	
Lysinibacillus						UFMG25
Microbacterium	UFMG61				UFMG53 UFMG56	
Obseumbacterium					UFMG60	
Ochrobactrum					CNPMS 2088	
Pantoea	UFMG83 UFMG54	UFMG38	UFMG67, UFMG74 UFMG93, CNPMS1934	UFMG7 UFMG36	UFMG55, UFMG59 CNPMS 2105	UFMG40
Pseudomonas	UFMG81				UFMG78	
Raoultella	UFMG69					
Rhizobium					UFMG71	
Serratia	UFMG85 UFMG48 UFMG44		UFMG41, UFMG42 UFMG43, UFMG94 CNPMS 2112			
Staphylococcus					UFMG77	
**Total per group**	**10**	**11**	**19**	**7**	**32**	**15**

ENDO – Endophyte bacteria; RIZO – bacteria isolated from rhizosphere.
