# Supplementary material for: Genomic and Phenotypic Insights Into the Potential of Rock Phosphate Solubilizing Bacteria to Promote Millet Growth in vivo
Source: Front Microbiol. 2021 Jan 7;11:574550. doi: 10.3389/fmicb.2020.574550 (PMC7817697; doi:10.3389/fmicb.2020.574550)
Supplement: Supplementary file 3 [file Table_3.pdf]

Table S3 – Clustering of bacteria based on profile of iron phosphate (Fe-P) solubilization in assays under agitation according the K-means analysis.

Bacterial genera	Group 1 (high solubilization)		Group 2 (medium solubilization)		Group 3 (low solubilization)	
	ENDO	RIZO	ENDO	RIZO	ENDO	RIZO
Acinetobacter					UFMG62	
Arthrobacter					UFMG63	
Bacillus			UFMG1923, CNPMS 2112		UFMG50, CNPMS 2106, UFMG49,	
Brevibacillus					UFMG57	
Burkholderia						
Curtobacterium			UFMG91			
Enterobacter			UFMG58, UFMG72, UFMG75, UFMG65		CNPMS 2084, UFMG84	UFMG31
Erwinia		UFMG04				
Flavobacterium			UFMG88			
Klebsiella		UFMG14, UFMG16 UFMG20, UFMG21 UFMG29, UFMG33	UFMG87, UFMG51	UFMG23, UFMG32, UFMG39	UFMG70	UFMG35
Lactococcus					UFMG66, UFMG76	
Lysinibacillus						
Microbacterium					UFMG61	
Obseumbacterium					UFMG60	
Ochrobactrum						
Pantoea	UFMG54, UFMG83	UFMG38	UFMG74, UFMG93, UFMG59, UFMG67	UFMG40	CNPMS 1934, CNPMS 2105	UFMG7
Pseudomonas					UFMG81	
Raoultella			UFMG69			
Rhizobium			UFMG71			
Serratia			UFMG42, CNPMS 2112, UFMG94		UFMG41, UFMG85, UFMG44	
Staphylococcus			UFMG90		UFMG92	
Total per group	**2**	**8**	**20**	**4**	**20**	**7**

ENDO – Endophyte bacteria; RIZO – bacteria isolated from rhizosphere.
