# Supplementary material for: Genomic and Phenotypic Insights Into the Potential of Rock Phosphate Solubilizing Bacteria to Promote Millet Growth in vivo
Source: Front Microbiol. 2021 Jan 7;11:574550. doi: 10.3389/fmicb.2020.574550 (PMC7817697; doi:10.3389/fmicb.2020.574550)
Supplement: Supplementary file 4 [file Table_4.pdf]

Table S4 – Clustering of bacteria based on profile of Araxá phosphate (AP) solubilization in assays with sessile cells according K-means analysis.

Bacterial genera	Group 1 (high solubilization)		Group 2 (medium solubilization)		Group 3 (low solubilization)	
	ENDO	RIZO	ENDO	RIZO	ENDO	RIZO
Acinetobacter					UFMG62<br>UFMG68	
Arthrobacter					UFMG96	
Bacillus					UFMG1923<br>CNPMS 2111	
Burkholderia		UFMG26				
Enterobacter	UFMG45		UFMG58	UFMG31,<br>UFMG30	UFMG84<br>UFMG75<br>UFMG72<br>UFMG65	UFMG8
Erwinia					UFMG4	
Flavobacterium					UFMG88	
Klebsiella	UFMG39	UFMG33<br>UFMG32<br>UFMG29<br>UFMG39<br>UFMG23	UFMG51,	UFMG2<br>UFMG20<br>UFMG21<br>UFMG35<br>UFMG14	UFMG87<br>UFMG79	UFMG1
Microbacterium					UFMG61	
Pantoea		UFMG40	UFMG67	UFMG7<br>UFMG38	CNPMS 1934<br>UFMG54<br>UFMG93<br>UFMG74<br>UFMG83	
Pseudomonas					UFMG81	
Raoultella					UFMG69	
Serratia	UFMG43<br>UFMG44		UFMG41		UFMG48<br>CNPMS 2112<br>UFMG85<br>UFMG42<br>UFMG94	
Staphylococcus					UFMG90	
**Total per group**	**4**	**7**	**4**	**9**	**27**	**2**

ENDO – Endophyte bacteria; RIZO – bacteria isolated from rhizosphere.
