# Supplementary material for: Genomic and Phenotypic Insights Into the Potential of Rock Phosphate Solubilizing Bacteria to Promote Millet Growth in vivo
Source: Front Microbiol. 2021 Jan 7;11:574550. doi: 10.3389/fmicb.2020.574550 (PMC7817697; doi:10.3389/fmicb.2020.574550)
Supplement: Supplementary file 5 [file Table_5.pdf]

Table S5: Prediction of CDS (coding sequences) related to P metabolism and phytohormones from the annotation made with Prokaryotic Genome Annotation System (PROKKA) and RAST (RAST-TK). The numbers represent the number of loci that each CDS has in the genomes

Bacteria		UFMG50	UFMG51	UFMG54	UFMG61	UFMG81	CNPMS2088
Genome size		6.14	6.54	4.27	3.76	6.07	5.5
Inorganic P uptake	Phosphate ABC transporter, periplasmic phosphate-binding protein PstS	1	1	2	1	1	1
	Phosphate transport ATP-binding protein PstB	2	2	2	1	1	1
	Phosphate transport system permease protein PstA	1	1	2	-	1	1
	Phosphate transport system permease protein PstC	1	1	2	1	1	1
	Low affinity inorganic phosphate transporter *pit*A	1	1	2	1	2	-
	Phosphate regulon transcriptional regulatory protein PhoB	1	2	1	-	2	1
	Phosphate regulon sensor protein PhoR	3	1	1	-	1	1
	ATP-binding pho regulon component PhoH	2	2	2	2	2	1
	Putative signaling at the cytoplasmic membrane PhoU	-	1	1	1	1	1
Organo phosphonate	Phosphonoacetate hydrolase PhnA	1	1	-	-	-	-
	Permease PhnB	1	1	-	-	-	-
	Phosphate-import ATP-binding protein PhnC	-	2	-	3	-	1
	Phosphate-import protein PhnD precursor	-	-	1	3	3	2
	Putative cryptic phosphonate transport system permease protein PhnE	-	2	2	4	2	2
	Putative transcriptional regulator PhnF	-	1	1	-	-	-
	Alpha-D-ribose 1-methylphosphonate 5-triphosphate synthase subunit PhnG	-	1	1	-	-	1
	Alpha-D-ribose 1-methylphosphonate 5-triphosphate synthase subunit PhnH	-	1	1	-	-	1
	Alpha-D-ribose 1-methylphosphonate 5-triphosphate synthase subunit PhnI	-	1	1	-	-	1
	Alpha-D-ribose 1-methylphosphonate 5-phosphate C-P lyase PhnJ	-	-	1	-	-	1
	Putative phosphonates utilization ATP-binding protein PhnK	-	1	1	-	-	1
	Alpha-D-ribose 1-methylphosphonate 5-triphosphate synthase subunit PhnL	-	1	1	-	-	1
	Ribose 1,5-bisphosphate phosphokinase PhnN	-	1	1	-	1	1
	Alpha-D-ribose 1-methylphosphonate 5-triphosphate diphosphatase PhnM	-	-	1	-	-	2
	2-aminoethylphosphonate transport system permease PhnU	-	-	-	-	1	1
	Putative 2-aminoethylphosphonate transport system permease protein PhnV	-	-	-	1	-	3
	Phosphonoacetaldehyde hydrolase	1	-	-	1	2	-
	Phosphonopyruvate hydrolase	1	-	-	-	-	-
	Phosphonatase *phn*XW	1				1	
Phosphatases	Alkaline phosphatase transcriptional regulatory protein	5	1	2	2	2	2
	Alkaline phosphatase	3	3	1	-	1	1
	Exopoliphosphatase *pp*X	1	2	1	1	1	-
	Polyphosphate kinase *ppk*	1	1	1	1	1	-
	Major phosphate-irrepressible acid phosphatase	-	2	1	-	-	-
