# Supplementary material for: Genomic and Phenotypic Insights Into the Potential of Rock Phosphate Solubilizing Bacteria to Promote Millet Growth in vivo
Source: Front Microbiol. 2021 Jan 7;11:574550. doi: 10.3389/fmicb.2020.574550 (PMC7817697; doi:10.3389/fmicb.2020.574550)
Supplement: Supplementary file 6 [file Table_6.pdf]

Table S6: Description of genes related to organic acid synthesis pathways containing the codes, KO, COG and EC of each genome.

Organic acids	UFMG50	UFMG81	CNPMS2088	UFMG61	UFMG51	UFMG54	Name	KO	COG	Definition	
Gluconic acid		1	1		1	1	gcd	K00117	COG499 3	quinoprotein glucose dehydrogenase	EC:1.1.5.2
	3		1				gdh	K00034	COG1028	glucose 1-dehydrogenase	EC:1.1.1.47
			1		1	2	gnl	K01053	COG3386	gluconolactonase	EC:3.1.1.17
							gnaD	K05308	COG4948	gluconate/galactonate dehydratase	EC:4.2.1.140
	3		1	3	1	2	kdgK	K00874	COG0524	2-dehydro-3-deoxygluconokinase	EC:2.7.1.45
					1	1	pqqB	K06136	COG: COG 1235	pyrroloquinoline quinone biosynthesis protein B	_
					1	1	pqqc	K06137	COG5424	pyrroloquinoline-quinone synthase	EC:1.3.3.11
		1			1	1	pqqD	K06138	_	pyrroloquinoline quinone biosynthesis protein D	_
		1			1	1	pqqE	K06139	COG0535	PqqA peptide cyclase	EC:1.21.98.4
	4	1	1	2	2	1	PGD, gnd, gntZ	K00033	COG0362 COG1023	6-phosphogluconate dehydrogenase	EC:1.1.1.44 1.1.1.343
	2		2		1	1	kduD	K00065	COG1028	2-dehydro-3-deoxy-D-gluconate 5-dehydrogenase	EC:1.1.1.127
	4	1		1	1	1	TC.GNTP	K03299	COG2610	gluconate:H+ symporter, GntP family	_
	1	1					gntR	K11476	COG1802	GntR family transcriptional regulator, gluconate operon transcriptional	_
		1	1		1	1	YliI	K21430	COG2133	Aldose sugar dehydrogenase	EC:1.1.5.-
2 - Keto-D-gluconic acid		1	1		1	1	E1.1.99.3A	K06151	COG2303	g luconate 2 -dehydro genase alpha chain	EC 1.1.99.3A
		1	1		1	1	gad	K06152	_	gluconate 2-dehydrogenase gamma chain	EC 1.1.99.3G
		1			1	1	ghrB	K00090	COG1052	2-keto-D-gluconate reductase - gluconate 2- dehydrogenase	EC 1.1.1.215
Formic acid	1	0	1	1		1	oxdD	K01569	COG2140	oxalate decarboxylase	EC:4.1.1.2
Glyoxylic acid (Glyoxylate cycle)	2	4	1	1	1	1	ACSS1_2, acs	K01895	COG0365	acetyl-CoA synthetase	EC:6.2.1.1
			1	1			PCCA, pccA	K01965	COG4770	propionyl-CoA carboxylase alpha chain	EC:6.4.1.3
	1	1	1	1	1	1	aceA	K01637	COG2224	isocitrate lyase	E4.1.3.1
	1	1	1	1	1	1	ACO, acnA	K01681	COG0065 COG1048	aconitate hydratase	EC:4.2.1.3
		1	1		1	1	acnB	K01682	COG1049	aconitate hydratase	EC:4.2.1.3
	2	1	2	1	2	1	CS, gltA	K01647	COG0372	citrate synthase	EC:2.3.3.1
	1	1	1	1	1	1	aceB, glcB	K01638	COG2225	malate synthase	EC:2.3.3.9
	2	1	1	1	1	1	MDH1	K00024	COG0039	malate dehydrogenase	EC:1.1.1.37
Glycolic acid		1			1	1	ghrB	K00090	COG1052	glyoxylate/hydroxypyruvate/2-ketogluconate reductase	EC:1.1.1.79; 1.1.1.81, 1.1.1.215
			2		2	2	ghrA	K12972	COG0111	glyoxylate/hydroxypyruvate reductase	EC:1.1.1.79; 1.1.1.81
		1			2	2	hpra	K00018		glycerate dehydrogenase	EC:1.1.1.29

	2		1				gyaR	K00015	COG1052	glyoxylate reductase	EC:1.1.1.26
					1	1	lldR	K14348	COG2186	L-lactate dehydrogenase operon regulator	_
	1						lldG	K00782	COG1556	L-lactate dehydrogenase complex protein	_
	1						lldF	K18929	COG1139	L-lactate dehydrogenase complex protein	_
	1						lldE	K18928	COG2048	L-lactate dehydrogenase complex protein	_
Latic acid	1						lctP	K03303	COG1620	lactate permeasse	_
	1			1	1		ldh	K00016	COG0039	L-lactate dehydrogenase	EC:1.1.1.27
	2						nhaC	K03315	COG1757	malate-2H(+)/Na(+)-lactate antiporter (mleN)	_
		1			1		nhaB	K03314	COG3067	Na+:H+ antiporter, NhaB family	_
		1	1	1	1	1	nhaA	K03313	COG3004	Na+:H+ antiporter, NhaA family	_
		1	1		1	1	acnB	K01682	COG1049	aconitate hydratase 2 / 2-methylisocitrate dehydratase	EC:4.2.1.3; 4.2.1.99
	1	1	1	1	1	1	acnA	K01681	COG0065 COG1048	aconitate hydratase	EC 4.2.1.3
Citric acid (Citrate cycle-first carbon oxidation)	2	1	2		2	1	CS, gltA	K01647	COG0372	citrate synthase	EC:2.3.3.1
	1	1	1	1	4	1	IDH1, IDH2, icd	K00031	COG0538	isocitrate dehydrogenase	EC:1.1.1.42
		1		1			prpB	K03417	COG2513	methylisocitrate lyase	EC 4.1.3.30
		1		1			prpD	K01720	COG2079	2-methylcitrate dehydratase	EC 4.2.1.79
			1	1	1	1	MDH1	K00025	_	malate dehydrogenase	EC:1.1.1.37
	1		1	1	1	1	fumA, fumB	K01676	COG1838	fumaratehydratase,class I	EC 4.2.1.2
			1	1	1	1	FrdA	K00244	COG1053	fumaratereductaseflavoprotein subunit	EC 1.3.5.4
			1	1	1	1	SDHA, SDH1	K00234	COG1053	Succinate dehydrogenase (ubiquinone)flavoprotein subunit	EC:1.3.5.1
	1	1	1	1	1	1	sucD	K01902	COG0074	succinyl-CoA synthetase alpha subunit	EC:6.2.1.5
Succinic acid (Citrate cycle - second carbon oxidation)	1	1	1				aarC, cat1	K18118	COG0427	succinyl-CoA:acetate CoA-transferase	EC:2.8.3.18
	1	1	1	1	1	1	DLST, sucB	K00658	COG0508	2-oxoglutarate dehydrogenase E2 component (dihydrolipoamide	EC:2.3.1.61
	3	3	1	1	1	1	DLD, lpd, pdhD	K00382	COG1249	dihydrolipoamide dehydrogenase	EC:1.8.1.4
	1	1	1	1	1	1	OGDH, sucA	K00164	COG0567	2-oxoglutarate dehydrogenase E1 component	EC:1.2.4.2
	2	1		1	1	1	mqo	K00116	COG0579	malate dehydrogenase (quinone)	EC:1.1.5.4
	1					1	korA, oorA, oforA	K00174	COG0674	2-oxoglutarate/2-oxoacid ferredoxin oxidoreductase subunit alpha	EC:1.2.7.3; 1.2.7.11
Acetic acid			1	1	1	1	poxB	K00156	COG0028	pyruvate dehydrogenase (quinone)	EC:1.2.5.1
		1	1	1	1	2	aldB	K00138	COG1012	aldehyde dehydrogenase	EC:1.2.1.-
